# An efficient method for indexing grazing-incidence X-ray diffraction data of epitaxially grown thin films

**DOI:** 10.1107/S2053273320001266

**Published:** 2020-04-02

**Authors:** Josef Simbrunner, Benedikt Schrode, Jari Domke, Torsten Fritz, Ingo Salzmann, Roland Resel

**Affiliations:** a Department of Neuroradiology, Vascular and Interventional Radiology, Medical University Graz, Auenbruggerplatz 9, Graz, 8036, Austria; b Institute of Solid State Physics, Graz University of Technology, Petersgasse 16, Graz, 8010, Austria; c Institute of Solid State Physics, Friedrich Schiller University Jena, Helmholtzweg 5, Jena, 07743, Germany; d Department of Physics, Department of Chemistry and Biochemistry, Centre for Research in Molecular Modeling (CERMM), Centre for NanoScience Research (CeNSR), Concordia University, 7141 Sherbrooke Street W., SP 265-20, Montreal, Québec H4B 1R6, Canada

**Keywords:** epitaxy, indexing, mathematical crystallography

## Abstract

Crystal structure identification of thin organic films entails a number of technical and methodological challenges. In particular, if molecular crystals are epitaxially grown on single-crystalline substrates a complex scenario of multiple preferred orientations of the adsorbate, several symmetry-related in-plane alignments and the occurrence of unknown polymorphs is frequently observed. In theory, the parameters of the reduced unit cell and its orientation can simply be obtained from the matrix of three linearly independent reciprocal-space vectors. However, if the sample exhibits unit cells in various orientations and/or with different lattice parameters, it is necessary to assign all experimentally obtained reflections to their associated individual origin. In the present work, an effective algorithm is described to accomplish this task in order to determine the unit-cell parameters of complex systems comprising different orientations and polymorphs. This method is applied to a polycrystalline thin film of the conjugated organic material 6,13-pentacene­quinone (PQ) epitaxially grown on an Ag(111) surface. All reciprocal vectors can be allocated to unit cells of the same lattice constants but grown in various orientations [sixfold rotational symmetry for the contact planes (102) and (102)]. The as-determined unit cell is identical to that reported in a previous study determined for a fibre-textured PQ film. Preliminary results further indicate that the algorithm is especially effective in analysing epitaxially grown crystallites not only for various orientations, but also if different polymorphs are present in the film.

## Abstract 2

A method is described for indexing grazing-incidence X-ray diffraction data of epitaxially grown thin films comprising various crystal orientations and/or polymorphs by measuring reciprocal-lattice vectors.

## 1. Introduction   

Crystal structure identification of thin films entails a number of technical and methodological challenges: (i) low scattering volumes translate into only a small number of observable diffraction peaks and (ii) under the presence of a substrate the crystallites grow in a preferred orientation (texture) (Birkholz, 2006 ▸). The situation becomes even more complex in the case of thin films formed by conjugated organic molecules. Their typical growth in crystal systems of low symmetry (in most cases monoclinic and triclinic), their tendency to polymorphism and thus the presence of several phases make crystal structure determination a difficult task (Tolan, 1999 ▸). Additionally, unknown polymorphs of organic materials are frequently observed within thin films only and cannot be determined independently via traditional methods like single-crystal diffraction (Jones *et al.*, 2016 ▸). On isotropic substrates, the crystallization of molecular materials typically results in fibre-textured films comprising crystallites that share a common fibre axis perpendicular to the substrate surface but are azimuthally randomly oriented (Witte & Wöll, 2004 ▸). The use of anisotropic substrates (like rubbed polymer surfaces or single-crystalline surfaces) or anisotropic preparation methods (like off-centre spin coating, dip coating or off-axis evaporation) can result in even more distinguished textures of the crystallites (Müller *et al.*, 1999 ▸; Brinkmann *et al.*, 2003 ▸; Qu *et al.*, 2016 ▸). In this context, particularly complicated cases include epitaxially grown molecular crystals on single-crystalline surfaces (Simbrunner *et al.*, 2011 ▸). There, multiple preferred orientations of the crystals relative to the substrate surface can occur together with several symmetry-related in-plane alignments of the crystallites. For example, for the epitaxial order of the conjugated organic molecule *para*-quaterphenyl on Au(111) surfaces two different preferred orientations have been found, which show 24 different in-plane alignments each (Müllegger *et al.*, 2003 ▸). For such films formed by epitaxially grown molecular crystals the identification and characterization of unknown polymorphs is a particularly challenging task (Dienel *et al.*, 2008 ▸).

**Figure 1:**
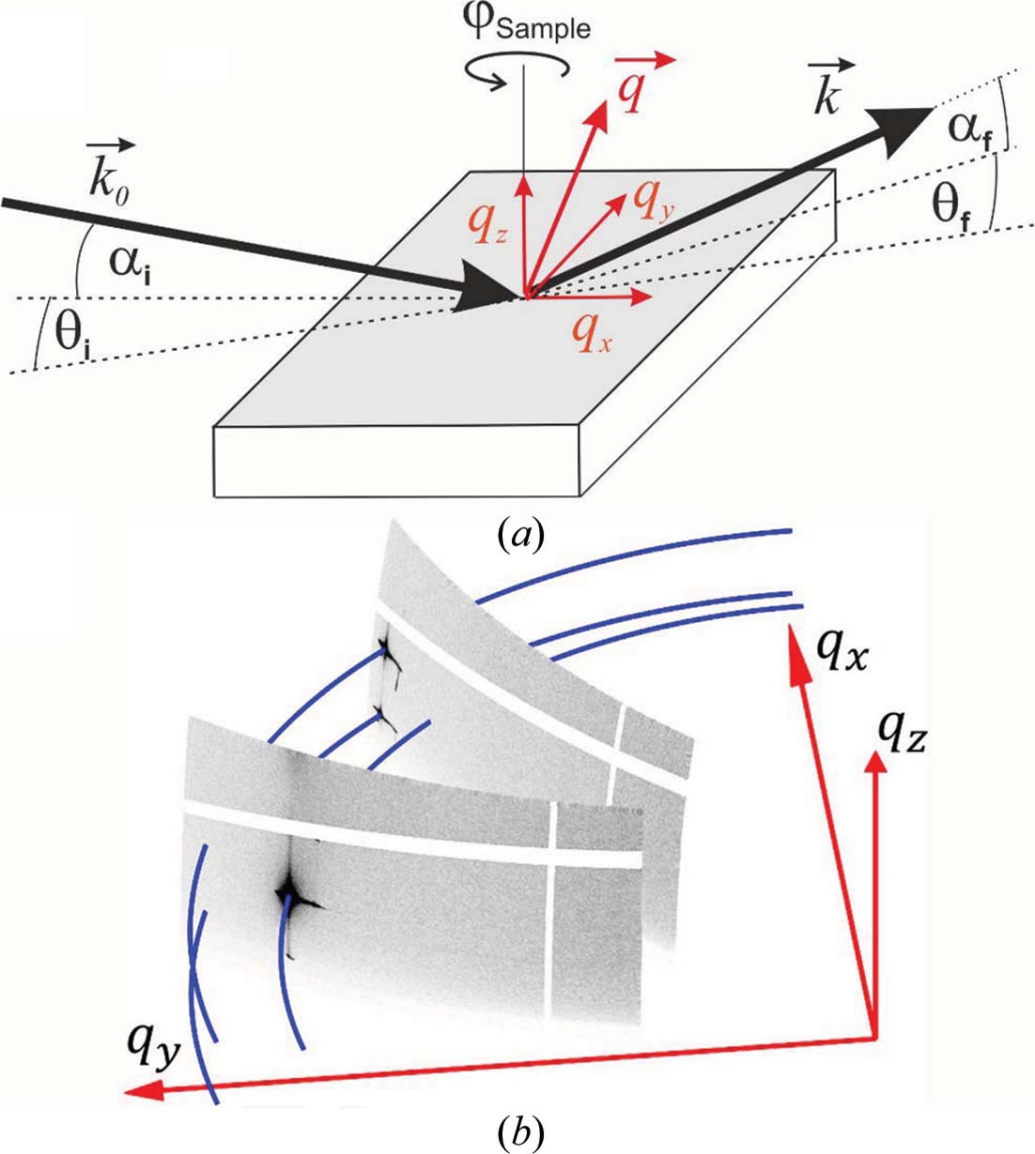
(*a*) Scattering geometry of a grazing-incidence X-ray diffraction experiment with a sample rotation around the angle φ_sample_. (*b*) Trajectory of reciprocal-lattice points during the rotation of the sample around the angle φ_sample_ along concentric circles (blue lines) and two individual reciprocal-space maps at defined angles φ_sample_.

Grazing-incidence X-ray diffraction (GIXD) is a well-established method for thin-film characterization (Schreiber, 2004 ▸). For non-fibre-textured films, in order to cover large volumes of the reciprocal space the sample has to be rotated around its surface normal during the GIXD experiment and at each rotation angle a reciprocal-space map has to be recorded (Fumagalli *et al.*, 2012 ▸; Schrode *et al.*, 2019 ▸). Fig. 1 ▸(*a*) gives the geometry of a GIXD experiment illustrating such a sample rotation. The primary X-ray beam defining the wavevector **k**_0_ and the scattered X-ray beam characterized by the wavevector **k** define the scattering vector **q** as **q** = **k**_0_ − **k**. The three components of the scattering vector *q_x_*, *q_y_* and *q_z_* can be calculated based on the geometries of primary and scattered X-ray beams by 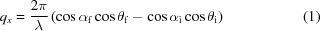


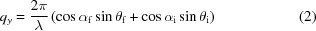


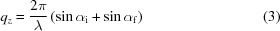
with *α*_i_ and *α*_f_ being the incident and exit angles of the primary and scattered beam (relative to the substrate surface), respectively, and θ_i_, θ_f_ are the in-plane scattering angles. Note that the orthogonal directions of *q_x_*, *q_y_* and *q_z_* are defined in the sample coordinate system. Fig. 1 ▸(*b*) illustrates (i) the path of reciprocal-lattice points during the sample rotation by φ_sample_, and (ii) cuts through reciprocal space which correspond to reciprocal-space maps recorded at two defined φ_sample_ angles.

Crystal structure solutions require the indexing of the diffraction pattern, *i.e*. the assignment of Laue indices to the observed Bragg peaks. In the monochromatic approach of peak indexing as it is commonly employed for single-crystal diffraction patterns, all three components of the scattering vectors are recorded and, therefore, all three components of the reciprocal-lattice vectors can be determined. Three linear­ly independent reciprocal-lattice vectors are sufficient to span the reciprocal lattice. Any further experimentally determined reciprocal-lattice vector then must fit into this specific reciprocal lattice. Since complete three-dimensional vectors are used, even the indexing of configurations with multiple lattices can be successfully achieved (Jacobson, 1976 ▸; Higashi, 1990 ▸; Powell, 1999 ▸; Breiby *et al.*, 2008 ▸; Gildea *et al.*, 2014 ▸; Dejoie *et al.*, 2015 ▸). Plenty of indexing methods have already been described over the last decades. Typically, a set of positions of recorded reflections is converted into reciprocal-space vectors. These vectors are then analysed for periodicity to determine the basis vectors. Differences between reciprocal-space vectors are calculated and accumulated in a histogram (Kabsch, 1988 ▸, 2010 ▸), or a fast Fourier transform (FFT) is used to search for periodicities (Steller *et al.*, 1997 ▸; Campbell, 1998 ▸; Leslie, 2006 ▸; Otwinowski *et al.*, 2012 ▸; Sauter *et al.*, 2004 ▸). For example, the autoindexing method incorporated into the software *MOSFLM* (Leslie, 1992 ▸) employs FFT autoindexing routines written by the Rossmann group at Purdue University (Rossmann & van Beek, 1999 ▸) and relies on the calculation of many difference vectors between diffraction maxima in reciprocal space (Kabsch, 1993 ▸). The Fourier analysis is systematically performed for about 7300 separate, roughly equally spaced directions. For each direction, the distribution of the corresponding Fourier coefficients is searched to locate the largest local maximum, and refinement by a local search procedure increases the accuracy. From these directions, a linearly independent set of three basis vectors of a real-space unit cell is then chosen.

In the approach by Duisenberg, periodicities are sought by projecting all observed reciprocal-lattice vectors onto the normal to the plane given by three randomly selected points (Duisenberg, 1992 ▸). This method was developed for difficult cases such as twin lattices, fragmented crystals and unreliable data. For such cases, Morawiec has developed another algorithm in which systematic combinations of three reciprocal-lattice vectors each are formed to search for periodicities of the calculated unit-cell volumes (Morawiec, 2017 ▸).

In the case of GIXD, as usually performed on fibre-textured films, however, only two components (of the total three) of the reciprocal-lattice vectors – namely *q_z_* and *q_xy_* – are available for the indexing procedure. In previous work, we have presented an algorithm for indexing such diffraction patterns, where the additional presence of a specular diffraction peak is being explicitly taken into account (Simbrunner *et al.*, 2018 ▸, 2019 ▸). Furthermore, we have described an algorithm to find the reduced cell and derived mathematical expressions which can be applied when reciprocal-space vectors are obtained.

In the present work, we now aim to formulate this indexing method for GIXD patterns obtained for rotated samples of non-fibre-textured films, which then provides all three components of the scattering vector. Also, in this case, the combination of the diffraction peaks obtained from GIXD with the specular diffraction peak(s) simplifies the indexing procedure considerably, so that different phases, different preferred orientations and different crystal alignments can be identified. Finally, our algorithm is applied to a film of 6,13-pentacene­quinone (C_22_H_12_O_2_, CAS No. 3029-32-1) epitaxially grown on a single-crystalline Ag(111) surface readily providing the unit-cell parameters of the film from GIXD data.

## 2. Method   

### 2.1. Fundamentals   

For the following mathematical treatise a crystal-fixed Cartesian coordinate system is assumed, where the *xy* plane runs parallel to the substrate surface; *a*, *b*, *c*, α, β and γ are the parameters of the (direct) unit cell.

If the (001) lattice plane is parallel to the substrate surface, the reciprocal-lattice vector **g** with its Laue indices *h*, *k* and *l* can be represented by the equation 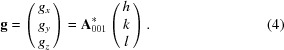
The matrix 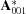 is given as 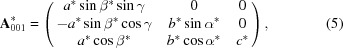
where 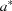 = 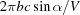, 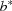 = 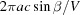, 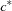 = 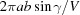, 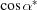 = 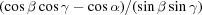 and 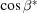 = 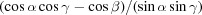 are the reciprocal cell parameters and *V* is the unit-cell volume, which can be explicitly written as 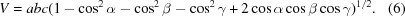
When the Laue condition **q** = **g** is fulfilled, diffraction can be observed. In real space, **A**_001_ characterizes the matrix of lattice vectors **a**_0_, **b**_0_ and **c**_0_, which is in the non-rotated system given by 
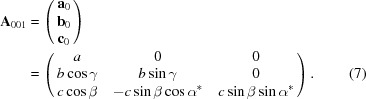
Equations (5) and (7) are connected via 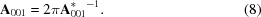
The volume *V* of the unit cell can be calculated by *V* = det(**A**_001_). If, however, the (001) plane is not parallel to the substrate surface, the reciprocal vector **g** can be expressed as 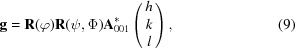
where **R**(φ)**R**(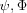) describes a general rotation. As explained previously (Simbrunner *et al.*, 2018 ▸), we prefer this notation. **R**(φ) performs a rotation in the *xy* plane counterclockwise by an angle 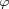 and is explicitly written as 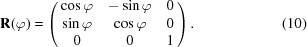
The rotation matrix **R** (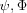) is explicitly written as 
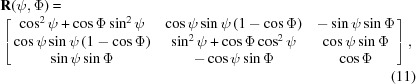
where 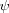 and 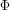 are the rotation angles. This rotation matrix was previously used for the mathematical formalism in GIXD experiments, where the composite component *q_xy_* is measured and, therefore, rotation in the *xy* plane can be neglected. It has been shown that, if the specular diffraction scan *q*_spec_ of the sample provides useful information on the orientation, it is preferable to write the rotation parameters as follows: 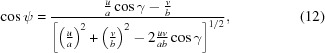


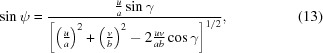


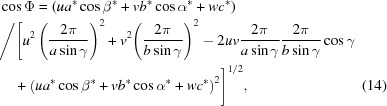
where *u*, *v* and *w* are the Miller indices of the contact plane (*uvw*) and thus the Laue indices of the specular scan. In the general case, *u*, *v* and *w* can be irrational numbers.

From equation (9), using equation (8), it follows that 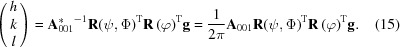
With 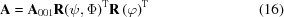
equation (15) can be rewritten as 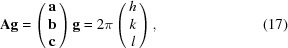
where **a**, **b** and **c** are the rotated unit-cell vectors with the relations 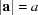, 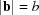, 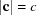, 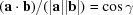, 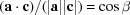 and 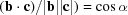. The explicit forms of these vectors are given in Table 1 ▸. Note that the *z* components are only a function of the respective Miller index and the specular scan *g*_spec_.

Thus, if three reciprocal vectors **g**_1_, **g**_2_ and **g**_3_ are given, the following relation holds: 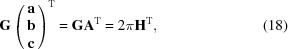
where 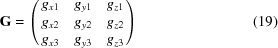
and (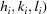 are the corresponding triples of Laue indices with 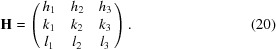



Equation (18) can be equivalently expressed as 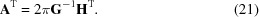
Furthermore, as 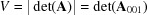, the following relation for the determinants of **G** and **H** is valid: 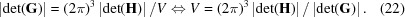
The unit-cell vectors must be solutions to all reciprocal vectors **g**_*i*_ which can be written as 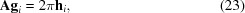
where
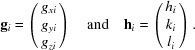
From equation (21) it can be deduced that 2π**G** ^−1^ **m**, the product of the inverse matrix of three reciprocal vectors with a vector **m**, consisting of a triple of arbitrary integers (*m*_1_, *m*_2_, *m*_3_), leads to a vector of the reduced cell (Niggli, 1928 ▸) if **m** matches (*h*_1_, *h*_2_, *h*_3_)^T^, (*k*_1_, *k*_2_, *k*_3_)^T^ or (*l*_1_, *l*_2_, *l*_3_)^T^. If a transformation matrix **N** exists so that **m** equals **N**(*h*_1_, *h*_2_, *h*_3_)^T^, **N**(*k*_1_, *k*_2_, *k*_3_)^T^ or **N**(*l*_1_, *l*_2_, *l*_3_)^T^ a vector of a superlattice is obtained. According to equation (22) it is favourable to select three reciprocal vectors whose matrix results in a determinant which is as small as possible but unequal to zero. The Buerger cell (Buerger, 1957 ▸) and subsequently the reduced cell is obtained by choosing the three shortest vectors which are not coplanar and whose scalar products with all reciprocal vectors yield integers.

### 2.2. Indexing algorithm   

We now suggest the following procedure for indexing an unknown crystalline system:

(i) Forming triplets of reciprocal vectors in all possible combinations, *i.e.* if *n* vectors are given these are
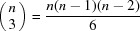
triplets (**g**_1_, **g**_2_, **g**_3_), where **g**_1_, **g**_2_ and **g**_3_ are any three reciprocal vectors. According to equation (22), if the triplet corresponds to a unit cell, the determinant of its matrix is indirectly proportional to the volume of this unit cell, or if the matrix of the corresponding Laue indices has a determinant >1, to some integer fraction of it. As many reciprocal vectors belong to one unit cell, they accumulate to discernible clusters. Importantly, a vanishing determinant strongly indicates that the reciprocal-lattice vectors (and the corresponding Laue indices) are linearly dependent and belong to the same crystalline system. If several unit cells are contained in the sample, an overlap of smaller volumes with fractions of larger volumes occurs. A feasible strategy results in gathering the reciprocal vectors of the largest volume and repeating the procedure for the remaining reciprocal vectors.

If the crystallites are characterized by the same unit cell and differ only in their rotational arrangement in the *xy* plane, this algorithm can only be used if the unit-cell volume is *a priori* known. Otherwise, three reciprocal vectors are chosen from different subgroups with identical pairs of *q_xy_* = (*q_x_* ^2^ + *q_y_* ^2^)^1/2^ and *q_z_*.

(ii) According to equation (19), the selected triplets of reciprocal vectors are combined into matrices. If they belong to the same system, their inverse matrices multiplied with the vectors of the corresponding Laue indices will result in the vectors of the unit cell [*cf*. equation (21)]. This can be achieved by multiplying the inverse matrices **G** ^−1^ with vectors 2π(*m*_1_, *m*_2_, *m*_3_)^T^, where the *m_i_* are systematically varied integers in a reasonable range (*e.g*. between −3 and 3). Then, lattice vectors of the unit cell and of its superlattices are obtained (Simbrunner *et al.*, 2018 ▸). The three shortest vectors which are not coplanar are chosen to obtain the Buerger cell. The as-obtained matrices contain the vectors **a**, **b** and **c** of the reduced unit cells which may have various orientations. If the reciprocal-space vectors are correctly combined, *i.e.* if they belong to the same system, the scalar product criteria (Niggli, 1928 ▸) are intrinsically fulfilled; otherwise they are useful to eliminate falsely combined vector triplets. The associated integers are the corresponding triples of Laue indices [see equation (18)]. If a contact plane exists and the specular scan *q*_spec_ can be measured, the triplets whose *z* components are (almost) integer multiples of 2π/*q*_spec_ can be assigned as possible solutions.

(iii) The tentative unit-cell matrices are multiplied with all reciprocal vectors. If the scalar products yield integers [*i.e.* the corresponding Laue indices according to equation (23)], the matrices and reciprocal vectors belong to the same system. Due to experimental imperfections, errors must be considered. For a system of reciprocal vectors, the unit cell with the smallest deviations from perfect integers will be chosen.

(iv) From the unit-cell matrix, the cell parameters and rotation parameters can be obtained. From equation (17) it follows that the sides of the unit cell are the magnitudes and its angles are the scalar products of the matrix vectors. The Niggli criteria for reduced cells demand that **a** ^2^ ≤ **b** ^2^ ≤ **c** ^2^ and that the angles are either acute (type I) or obtuse (type II) (Niggli, 1928 ▸). Therefore, the vectors have to be designated
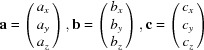
accordingly. Furthermore, the angles must be adopted. By multiplying one vector with −1 two angles change to their complementary ones, *e.g.* for **a** → −**a** we obtain β → π − β and γ → π − γ. The rotation angles φ, ψ and ϕ can be obtained by using the equations provided in Appendix *A* [App appa].

(v) According to equation (21) the unit-cell vectors can be calculated from every linearly independent triple of reciprocal-lattice vectors of the same system. This redundancy can be used to determine mean values and standard deviations of the unit-cell parameters. Alternatively, the matrix of the unit-cell vectors may be optimized in real space (see Appendix *B* [App appb]) and in reciprocal space (see Appendix *C* [App appc]). In a last step, the lattice parameters may be optimized with respect to the lengths *q_xyz_* and the components *q*_*z*_ of the reciprocal-space vectors, which are independent of the rotation angle φ. This can be accomplished analytically (see Appendix *D* [App appd]).

## 3. Example: 6,13-pentacene­quinone (PQ) on Ag(111)   

We now apply the above methodology to an epitaxially grown film of the conjugated organic molecule 6,13-pentacene­quinone (PQ, C_22_H_12_O_2_, CAS No. 3029-32-1) on an Ag(111) surface. PQ was purchased from Sigma Aldrich (purity 99%) and purified via vacuum sublimation before usage. Substrate preparation and film preparation were conducted in an ultra high vacuum (UHV) chamber with a base pressure of 1 × 10^−8^ Pa. Before thin-film deposition, the substrate surface was cleaned by repeated cycles of Ar^+^ sputtering at an energy of 700 eV and angles of ±45° to the sample normal, followed by thermal annealing at 770 K for 30 min. Surface quality was confirmed by low-energy electron diffraction (LEED). PQ was deposited by thermal evaporation from a quartz crucible at a constant temperature of 480 K for 60 min with the deposition time controlled by a shutter, resulting in an approximate film thickness of 10 nm. During deposition, the substrate was kept at room temperature and the chamber pressure increased to 4 × 10^−7^ Pa. A top layer of aluminium tris-(8-hy­droxy­quin­oline) (Alq3, C_27_H_18_AlN_3_O_3_, CAS No. 2085-33-8), known to grow amorphously, was deposited from a quartz crucible at a constant temperature of 550 K for 55 min to keep the PQ crystals free from environmental influences and to reduce beam damage during the X-ray diffraction experiments. Alq3 was obtained from Sigma Aldrich at sublimed grade with a purity of 99.995% trace metal basis and was used without further purification.

The sample was first investigated by specular X-ray diffraction using in-house equipment and then by GIXD using synchrotron radiation. Specular X-ray diffraction was performed on a PANalytical Empyrean system using a sealed copper tube together with an X-ray mirror for monochromatization and a PIXcel3D detector operating in scanning line mode (255 channels). Measured data were converted to reciprocal space using 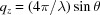, with θ being half of the scattering angle 2θ and λ = 1.5406 Å. GIXD measurements were performed at the XRD1 beamline at the Elettra Synchrotron, Trieste (Italy), using a wavelength of 1.4000 Å. Diffracted intensity was recorded with a stationary Pilatus 2M detector (Dectris) with a sample-to-detector distance of about 200 mm. The primary X-ray beam was slightly offset from the centre of the detector to allow for simultaneously recording both the right- and the left-hand side of the reciprocal-space map, but to avoid missing peaks due to detector gaps typical for the Pilatus system. The calibration of the setup (to obtain exact values for sample–detector distance, position of the primary beam on the detector, detector inclinations *etc*.) was performed by measuring polycrystalline lanthanum hexa­boride (LaB_6_) (Black *et al.*, 2010 ▸) in a capillary. The incident angle α_i_ for the thin-film measurement was set to 0.7° to reduce the footprint of the beam on the sample and, thus, enable data evaluation with higher accuracy. The sample was rotated around its surface normal [*cf*. Fig. 1 ▸(*b*), φ_sample_] during the GIXD measurement recording 180 images, with therefore each exposure integrating 2° in φ_sample_ of the azimuthal rotation. Note that the angle φ_sample_ is the experimentally used sample azimuth, while the angle φ is used to describe a rotation of the unit cell in the *xy* plane in the counterclockwise direction [*cf*. equations (9)–(10)].

**Figure 2:**
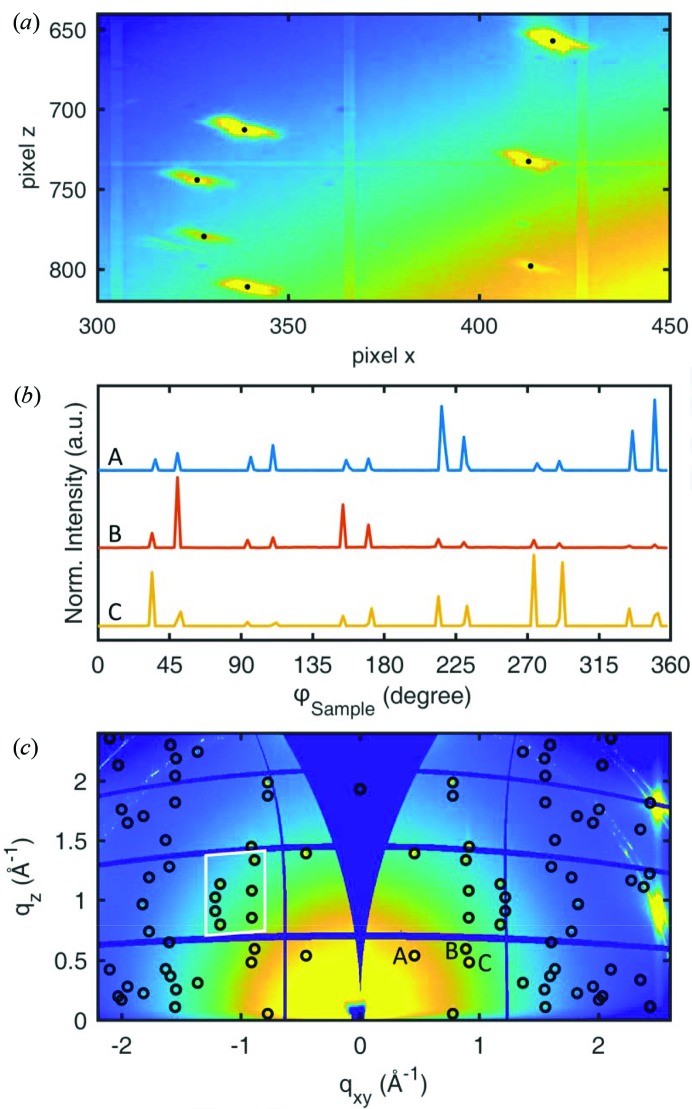
(*a*) Section of the integrated pixel image with black markers indicating the peak positions obtained from the fitting process. (*b*) Intensities of peaks A, B and C of (*c*) as a function of the sample rotation angle φ_sample_. (*c*) Integrated reciprocal-space map overlaid with the calculated peak positions of the determined crystal structure. The white box indicates the approximate section visualized in (*a*).

To determine the peak positions from the diffraction images, the following process was performed: in a first step, all diffraction images recorded during the 360° sample rotation were summed up pixel by pixel resulting in a single image showing the overall integrated diffraction information. The peak positions were then fitted using a two-dimensional Gaussian function with a background plane, giving the pixel position of each peak [*cf*. Fig. 2 ▸(*a*)].

To determine the peak positions as a function of the sample rotation, the intensity at the obtained peak positions was monitored throughout the 180 separate data files which translates into a curve representing intensity versus φ_sample_. Three curves are given in Fig. 2 ▸(*b*), where due to both the symmetry of the crystal structure and that of the substrate, several peaks are then observed for a given set of Miller indices. Most peaks show a full width at half-maximum (as a measure of in-plane mosaicity) of about 2°. Their positions were determined by fitting the corresponding part of the curve with a one-dimensional Gaussian function with linear background. In the next step, a small area around the peak position of the summed image was fitted with a two-dimensional Gaussian function in the specific data file corresponding to the rounded φ_sample_ value where the peak maximum was observed. These pixel positions together with the corresponding (unrounded) φ_sample_ values were used to convert the data into reciprocal space using source code provided by the software package *GIDVis* (Schrode *et al.*, 2019 ▸). No refraction corrections were included during the conversion. As mentioned above, the diffraction pattern was recorded simultaneously at the right-hand side (RHS) as well as at the left-hand side (LHS) of the detector. Therefore, the above-described data evaluation can be applied separately to the data from the RHS and the LHS, since each detector side (apart from the detector gaps) contains the same information for the data analysis, resulting in individual solutions for RHS and LHS data. Large differences between peak positions obtained from the LHS and RHS would indicate a sample misalignment, but were not observed here.

**Figure 3:**
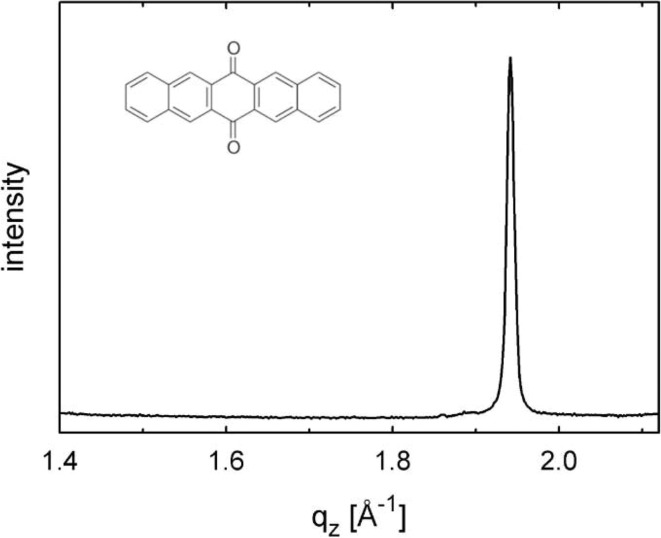
Specular X-ray diffraction of epitaxially grown pentacene­quinone crystals on an Ag(111) surface deposited with a nominal thickness of 10 nm. The chemical structure of the molecules is given in the inset.

**Figure 4:**
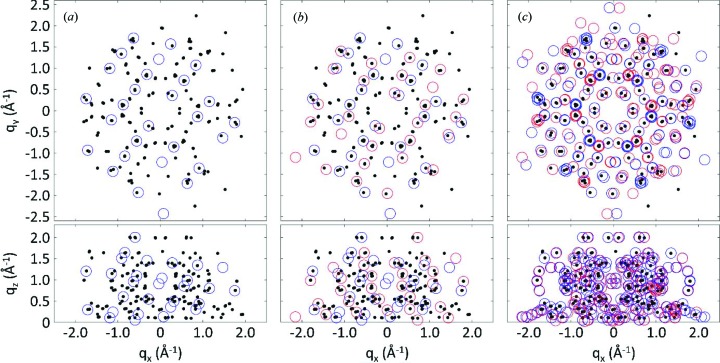
Positions of X-ray diffraction peaks (black) of pentacene­quinone crystals grown on an Ag(111) surface obtained from rotating GIXD experiments. Top: *q_y_*/*q_x_* positions of the diffraction peaks; bottom: *q_z_*/*q_x_* positions. (*a*) Indexing (blue circles) of a single type of epitaxially oriented crystals grown with the (102) plane parallel to the substrate surface; (*b*) a second type of crystals grown with the (102) contact plane is indexed (red circles); (*c*) indexing of all 12 types of epitaxially oriented crystals.

The specular X-ray diffraction pattern shows dominant features of the Ag(111) substrate and a clear diffraction peak which is assigned to PQ crystals (compare Fig. 3 ▸). According to our notation the position of this peak is *q*_spec_ = *q_z_* = 1.942 Å^−1^. The GIXD experiments gave 227 and 279 reciprocal-lattice vectors with the three components *q_x_*, *q_y_* and *q_z_* on the RHS and LHS, respectively. The different number is due to some peaks falling into the detector gaps at one detector side only. The reciprocal-lattice vectors could be split into 31 groups with up to 12 related pairs of *q_xy_* = (*q_x_* ^2^ + *q_y_* ^2^)^1/2^ and *q_z_*. Therefore, it could be concluded that there are 12 different in-plane alignments of the crystallites which are oriented with the same contact plane (*uvw* or −*u*−*v*−*w*). Three reciprocal vectors from different groups were then systematically combined, and the matrix was formed according to equation (16). Note that the determinant of the matrix is indirectly proportional to the volume of the unit cell [see equation (22)], and a determinant of zero expresses that linearly dependent lattice vectors have been chosen. In a next step the inverse matrix was multiplied with vectors of systematically varied integers, and the three shortest lattice vectors were chosen (Buerger cell) for a guess of a unit cell. If the *z* components of these tentative lattice vectors were integer multiples of 2π/*q*_spec_ and the Niggli criteria were fulfilled, they were assigned as possible solutions. The obtained integers could be assigned as the Miller indices *uvw* = (102) or (102) of the contact plane which finally gives the preferred orientation of the PQ crystals relative to the substrate surface. In a next step the tentative lattice matrix was multiplied with all reciprocal vectors of the other groups. If the lattice matrix was indeed a solution, triples of Laue indices could be assigned to the associated reciprocal vectors, according to equation (23). In a further step, the matrix of the unit-cell vectors was optimized (see Appendices *B* [App appb] and *C* [App appc]). On request, the used code can be provided. Fig. 4 ▸(*a*) shows the result of the indexing procedure for a single type of epitaxially aligned crystallites with the contact plane *uvw* = (102) for the RHS data; the assigned peaks are marked with blue circles.

For each solution the lattice vectors **a**, **b** and **c** were used to determine the respective lattice constants, the contact plane [*uvw* = (102) or (102)] and the rotation angle φ (see Appendix *A* [App appa]). As φ can be independently calculated from each of the three lattice vectors, the accuracy can be checked. In our case, the mean error was about 0.15%. Rotation angles between the different solutions were obtained in steps of Δφ = 60.00° (±0.26°) for the (102) contact plane and Δφ = 60.00° (±0.94°) for the (102) contact plane. This clearly reflects the symmetry of the Ag(111) surface. By repeating the procedure, all reciprocal vectors could be allocated to unit cells with the same lattice constants but various orientations.

In the case of Fig. 4 ▸(*b*) two types of epitaxially aligned crystallites are indexed with contact planes (102) and (102), denoted by blue and red circles, respectively. Fig. 4 ▸(*c*) gives indexing of all diffraction peaks by the two contact planes (102) and (102), each showing six different types of in-plane alignment of the crystallites. We found the following epitaxial relationships: (111)_Ag_ || ± (102)_PQ_; the *b* axis [010] of PQ in (102) orientation is rotated by −7° (*i.e.* clockwise) with respect to the 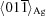 directions and the *b* axis [010] of PQ in (102) orientation is rotated by +7° (*i.e.* counterclockwise) with respect to the 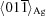 directions. An evaluation of the data from the LHS and RHS of the detector was then performed. As expected, no significant differences in the unit-cell vectors (including contact planes, epitaxial relationships and lattice constants) were found. The lattice constants were determined by averaging over all 24 sets of lattice constants. We then obtained *a* = 5.059 ± 0.012, *b* = 8.097 ± 0.026, *c* = 8.916 ± 0.032 Å, α = 91.64 ± 0.24°, β = 92.95 ± 0.56°, γ = 94.17 ± 0.23°, *V* = 363.5 Å^3^.

In a last step, we optimized the lattice parameters with respect to the vector lengths *q_xyz_* and the components *q*_*z*_ (see Appendix *D* [App appd]) and obtained the following values: *a* = 5.063, *b* = 8.091, *c* = 8.916 Å, α = 91.61°, β = 92.92°, γ = 94.13°, *V* = 363.6 Å^3^. These parameters differ only slightly from the values given above and the error function *E_xyz,z_* decreases only minimally from 0.0113 to 0.0112 Å^−1^.

**Figure 5:**
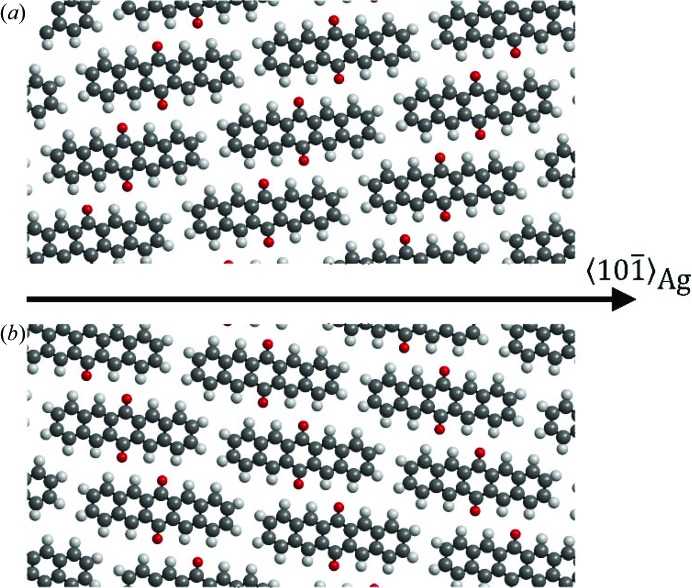
Two different epitaxial alignments of pentacene­quinone crystals on the Ag(111) surface, (*a*) with the (102) plane and (*b*) with the (102) plane parallel to the surface. The *b* axis is rotated by ±7° with respect to the high-symmetry direction on the Ag(111) surface.

The expected peak positions of this solution are plotted together with the two-dimensional reciprocal-space map in Fig. 2 ▸(*c*). A three-dimensional representation of the experimental data and the expected peak positions is given in Fig. 4 ▸. In both, a good agreement is observed. The lattice constants we obtain by following this protocol are in excellent agreement with the crystal structure of PQ reported for thin films grown on a highly oriented pyrolytic graphite (HOPG) surface (Simbrunner *et al.*, 2018 ▸). Furthermore, comparison of experimental with expected peak intensities shows good agreement. Therefore, the known crystal structure solution can be used to develop a model of the arrangement of PQ molecules with respect to the Ag(111) surface. Fig. 5 ▸(*a*) shows the situation of the (102) contact plane and a *b*-axis rotation angle of +7° with respect to the high-symmetry direction of the silver surface. Nearly completely flat-lying molecules are found which are rotated with respect to the high-symmetry silver directions. Adjacent molecules are slightly slipped to form short contacts between neighbouring oxygen and hydrogen atoms, which highlights the role of hydrogen bonding in the formation of the PQ crystal structure. In turn, for the (102) contact plane a *b*-axis rotation angle of −7° with respect to the silver high-symmetry direction [*cf*. Fig. 5 ▸(*b*)] is found, *i.e*. a rotation of the molecules in the opposite direction.

## 4. Discussion   

We regard our algorithm for the analysis of X-ray diffraction patterns of thin films via rotating the sample to determine reciprocal-lattice vectors advantageous for the following reasons: (i) the lattice vectors of the involved unit cells and their orientation can be determined simultaneously. The method is suitable for implementation of (semi)automatic processing. (ii) Only a few reciprocal vectors are required. Theoretically, only three vectors are sufficient to determine the parameters and orientation of the unit cell if the determinant of the matrix of the corresponding Laue indices is ±1. (iii) Indexing is possible even if crystals with different crystallographic unit cells and orientations are present. Depending on measurement accuracy and available boundary conditions, about six to eight related reciprocal vectors may be sufficient for a correct assignment to the corresponding unit cell. The knowledge of a contact plane as determined by available specular diffraction data can be of considerable help for selecting the proper unit-cell vectors. (iv) No previous knowledge of the structure is necessary, and the intensities of the various reflections are not required. For symmetry considerations, however, the diffraction intensities must be included.

We note that our method of combination of three reciprocal-lattice vectors has been suggested before (Duisenberg, 1992 ▸; Morawiec, 2017 ▸). In both cases this approach is used for solving difficult cases in single-crystal diffractometry such as twin lattices, fragmented crystals and unreliable data. Duisenberg takes the end points of three observed reflection vectors to build the normal to the plane formed by any three of these points. Then all observed points are projected onto this normal. This helps in eliminating spurious vectors which do not belong to the direct cell. Morawiec combines three reciprocal-lattice vectors to calculate the corresponding unit-cell volumes and searches for periodicities to select the vectors that support the definite parallelepiped. This procedure is related to the algorithm we suggest for selecting the proper reciprocal-lattice vectors. In our case, however, the direct unit cell is determined first, and then further reciprocal-lattice vectors are selected.

Immediately switching into real space for finding the unit-cell vectors is advantageous as several possible criteria and boundary conditions may exist for reducing possible solutions: (i) unit-cell volume and parameters, (ii) scalar product criteria (Niggli conditions) and (iii) including information from the specular scan if a contact plane is experimentally determined. To reduce the errors due to experimental imperfections, the lattice vectors of one unit cell are fitted before calculating the cell parameters and orientation angles. This is accomplished by analytically minimizing error functions, which can be defined both for reciprocal and real space.

Finally, our methodology allows the direct analytical determination of the orientation parameters, *i.e.* the angles ϕ, ψ (or the Miller indices *u*, *v*, *w* of the contact plane) and φ of the rotation matrices. These angles refer to the chosen reference system.

If a very large number of reciprocal vectors are obtained, alternative algorithms may be advantageous. The existing autoindexing method incorporated in *MOSFLM* (Leslie, 1992 ▸) – FFT autoindexing routines written by the Rossmann group at Purdue University (Rossmann & van Beek, 1999 ▸) – relies on the calculation of many difference vectors between diffraction maxima in reciprocal space (Kabsch, 1993 ▸). Preliminary results, however, show that our method is especially effective in analysing epitaxially grown crystallites not only with various orientations but also with various polymorphs.

## 5. Conclusion   

In this work, we present an algorithm for indexing GIXD diffraction patterns obtained with monochromatic radiation, where three-dimensional reciprocal-lattice vectors are determined as is done in single-crystal diffraction experiments. Our method is particularly advantageous if the number of reflections is relatively small or the sample is inhomogeneous and consists of various crystal lattices or orientations, as is commonly found for thin films grown on single-crystalline substrates. For easy access to epitaxial relationships the lattice constants of the involved unit cells and the parameters of the orientation matrix can be determined simultaneously.

## Figures and Tables

**Table 1:** Unit-cell vectors for the parameters 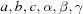, the Laue indices *hkl* and the Miller indices *uvw* and including the specular scan (*g*_spec_) for the non-rotated (*a*) and the rotated (*b*) case 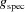
 = 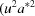 + 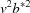 + 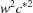 + 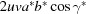 + 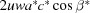 + 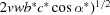.

(*a*) *Non-rotated* case (*u* = *v* = 0):		
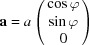		
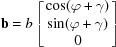		
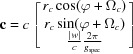	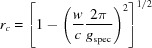	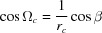
(*b*) *Rotated* case:		
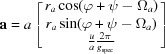	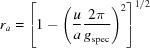	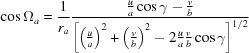
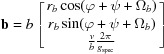	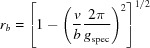	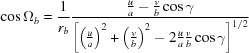
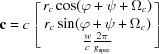	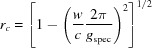	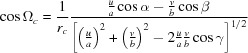
	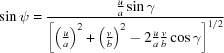	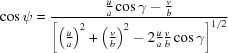

## Appendix A Determining the rotation angles ψ, ϕ and φ

The rotation parameters ψ and ϕ and the parameters Ω_*a*_, Ω_*b*_ and Ω_*c*_ can be preferably obtained by using the following equations, which can easily be derived from the expressions in Table 1 ▸ and equation (14):

Note that two special cases must be considered:

(i) If *a_z_* < 0 (*i.e. u* < 0) then  → 2π − ψ [as  is in the interval [π, 2π], see equation (13)].

(ii) If *a_z_* = 0 and *b_z_* = 0 (*i.e. u* = *v* = 0) then  = 0,  = 0, Ω_*a*_ = 0, Ω*_b_* =  and cos Ω_*c*_ = cos(β)/*r_c_*.

If the ratios *a_z_*/*b_z_* = *u*/*v*, *a_z_*/*c_z_* = *u*/*w* and *b_z_*/*c_z_* = *v*/*w* are rational numbers, one can conclude that a contact plane (with the Miller indices *uvw*) exists. If the specular diffraction peak is known, the indices can be directly calculated from *u* = (*a_z_*/*a*)(*g*_spec_/2π), *v* = (*b_z_*/*b*)(*g*_spec_/2π) and *w* = (*c_z_*/*c*)(*g*_spec_/2π).

From Table 1 ▸ the following expressions for the phase φ can be deduced:

As φ covers the range between 0 and 2π, equations (29) and (30) must be considered. This can be expressed in the following way:

Analogously, the following relations are valid:

As φ can be independently calculated from the lattice vectors **a**, **b** and **c**, the accuracy of the result can be checked and a mean value of φ can be determined.

## Appendix B Optimizing the matrix of the unit-cell vectors in real space

From equation (23) the error regarding the unit-cell vector **a** with respect to *N* associated reciprocal-lattice vectors **g**_*i*_ and the corresponding Laue indices *h_i_* can be expressed as

*E_a,N_* becomes minimal if

is fulfilled. From this condition the following relations can be derived:

where

Analogously, the error functions for the cell vectors **b** and **c** with respect to the measured reciprocal-lattice vectors **g**_*i*_ and the corresponding Laue indices *k_i_* and *l_i_*, respectively, can be expressed as

Then, *E_b,N_* and *E_c,N_* become minimal for

## Appendix C Optimizing the matrix of the unit-cell vectors in reciprocal space

In analogy to equations (4) and (9) the reciprocal-lattice vectors **g**_*i*_ can be written as

where *a_x_**, *a_y_**, *a_z_**, *b_x_**, *b_y_**, *b_z_**, *c_x_**, *c_y_**, *c_z_** are the components of the reciprocal-space vectors, and *h_i_*, *k_i_*, *l_i_* are the corresponding Laue indices. Then, for *N* reciprocal-space vectors the error function *E_x,N_* can be expressed as

*E_x_* becomes minimal if

is fulfilled. From this condition the following relations can be derived:

where

Analogously, the error functions for the cell vectors **b** and **c** with respect to the measured reciprocal-lattice vectors **g**_*i*_ and the corresponding Laue indices *h_i_*, *k_i_* and *l_i_* can be expressed as

Then, *E_y,N_* and *E_z,N_* become minimal for

The components of the unit cell in real space can be calculated from the following expression:

## Appendix D Analytical optimization of the unit-cell parameters with respect to *q_xyz_* and *q_z_*

If the Laue indices are determined and the unit-cell parameters are calculated by evaluating the reciprocal vectors as described above, in a last step they may be optimized with respect to the measured vector length *q_xyz_* and the component *q_z_*, which are phase-invariant. This can be accomplished analytically by considering small errors and first-order correction. It is convenient to use the reciprocal cell parameters and minimize the quadratic error function *E_xyz,z_*:

where

(*q_xyz,i_*, *q_z,i_*) are the measured and (*g_xyz,i_*, *g_z,i_*) are the calculated peak positions of the *i*th reflection, *n* is the number of reflections and  are the correction terms.  becomes minimal if

is fulfilled. From this condition the following relation can be derived:

where the matrix contains the inner products of the vectors

Furthermore, for the length *g_xyz,i_*, the out-of-plane component *g_z,i_* of the *i*th reciprocal vector and the specular scan *g*_spec_ the following expressions are valid:

From equation (54) the following derivatives of *g_xyz,i_* can be obtained:

From equation (55), using equation (56), the following derivatives of *g_z,i_* result:

From equation (52) the correction terms can be determined and added to the corresponding reciprocal cell parameters to obtain the new values. For larger errors it is advantageous to repeat this procedure. The cell parameters in real space are finally obtained by using their relations with the reciprocal-lattice parameters [*c.f.* equation (5)].
